# Precise control of coupling strength in photonic molecules over a wide range using nanoelectromechanical systems

**DOI:** 10.1038/srep24766

**Published:** 2016-04-21

**Authors:** Han Du, Xingwang Zhang, Guoqiang Chen, Jie Deng, Fook Siong Chau, Guangya Zhou

**Affiliations:** 1 Department of Mechanical Engineering, National University of Singapore, 9 Engineering Drive 1, Singapore 117576; 2 Institute of Materials Research and Engineering, A *STAR (Agency for Science, Technology, and Research), 2 Fusionopolis Way, Innovis, #08-03, Singapore 138634.

## Abstract

Photonic molecules have a range of promising applications including quantum information processing, where precise control of coupling strength is critical. Here, by laterally shifting the center-to-center offset of coupled photonic crystal nanobeam cavities, we demonstrate a method to precisely and dynamically control the coupling strength of photonic molecules through integrated nanoelectromechanical systems with a precision of a few GHz over a range of several THz without modifying the nature of their constituent resonators. Furthermore, the coupling strength can be tuned continuously from negative (strong coupling regime) to zero (weak coupling regime) and further to positive (strong coupling regime) and vice versa. Our work opens a door to the optimization of the coupling strength of photonic molecules *in situ* for the study of cavity quantum electrodynamics and the development of efficient quantum information devices.

Interactions between optical micro/nano resonators, which are strongly coupled to each other through their evanescent fields, have been intensively investigated for both their fundamental physics and potential applications^1^,^2^,^3^,^4^,^5^,^6^,^7^,^8^,^9^,^10^. Analogous to chemical molecules formed by coupled atoms, coupled optical micro/nano resonators are known as photonic molecules^11^,^12^. The coupling strength between resonators of photonic molecules, which characterizes their interaction, is a key parameter^8^,^13^,^14^,^15^,^16^,^17^,^18^. Precise control of coupling strength enables the entanglement generation in quantum-emitter-embedded photonic molecule systems^13^,^19^, improvement of single photon statistics in single photon devices 14, realization of spontaneous symmetry breaking (SSB)^15^ and Josephson phenomenon^16^,^17^ in photonic molecules, and many others. However, due to fabrication imperfections, fine control of coupling strength is hard to achieve. Although several methods including laser non-thermal oxidation and water micro-infiltration have been developed for post-fabrication compensation of the coupling strength, they are however irreversible, hard to control and have limited accuracy and tuning ranges^20^,^21^. The precise control of coupling strength over a wide range still presents a significant challenge.

Nanoelectromechanical systems (NEMS), which combine electrical and mechanical functionalities at the nanoscale, have emerged as a versatile tool for tuning photonic nanostructures^22^. Currently, NEMS tunable photonic devices for example tunable photonic crystals (PhCs)^23^,^24^,^25^,^26^, tunable lasers^27^, and phase modulators^28^,^29^ have been reported. Here, to the best of our knowledge, we demonstrate experimentally for the first time the use of monolithically integrated NEMS actuators to precisely control the coupling strength of photonic molecules by controlling the relative lateral displacements of two mutually coupled PhC nanobeam cavities (PhC nanobeam photonic molecules). Compared with photonic molecules based on microdisks or 2D PhC nanocavities, PhC nanobeam photonic molecules possess both high quality factor to modal volume ratios (Q/V) and small footprints^30^. Moreover, the two suspended nanobeams are spatially separated from each other, with one fixed and the other movable and integrated with NEMS actuators. Thus, the relative offset between them can be precisely controlled without modifying the nature of their constituent cavities, which is difficult to be realized in microdisks^12^ and 2D PhC photonic molecules^20^,^21^,^31^,^32^,^33^,^34^,^35^. We find from our experiments that the coupling strength can be continuously tuned from a negative value to a positive value with a control precision of a few GHz over a range of several THz, and that the tuning is completely reversible. This means that the photonic molecules can be dynamically tuned from a strong coupling regime to a weak coupling regime, and vice versa. Meantime, the parity of each supermode is able to be inversed when the sign of coupling strength inverses. Our work unfolds the capability to optimize the coupling strength in photonic molecules *in situ* that can be used in a broad range of applications such as the study of cavity quantum electrodynamics and the development of efficient quantum information devices.

## Results

### NEMS controlled PhC nanobeam photonic molecules

**Figure 1:**
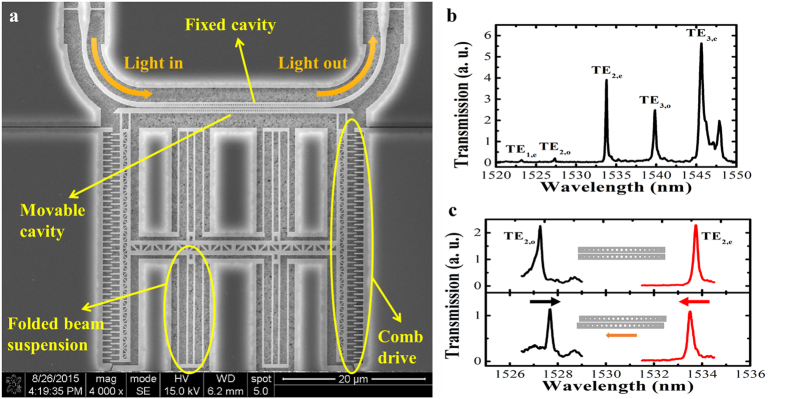
(**a**) SEM image of the NEMS controlled PhC nanobeam photonic molecule; (**b)** The transmission spectra of PhC nanobeam photonic molecule. Five modes are observed, namely TE_1,e_, TE_2,o_, TE_2,e_, TE_3,o_, TE_3,e_, corresponding to the 1^st^ even-like mode, the 2^nd^ odd-like mode, the 2^nd^ even-like mode, the 3^rd^ odd-like mode and the 3^rd^ even-like mode (FDTD simulations are used to identify the modes); (**c**) Illustration of mode splitting width change of PhC nanobeam photonic molecule due to a lateral displacement of the movable nanobeam cavity.

Our device is fabricated on a SOI wafer, following a series of standard nano fabrication processes. (see Methods for details of the fabrication processes). Figure 1a shows the SEM image of the NEMS tunable PhC nanobeam photonic molecule device. As can be seen, one of the PhC nanobeam photonic molecule cavities is fixed and light is launched into and coupled out from this cavity through suspended Si nanowire waveguides. The other cavity is movable and integrated with NEMS comb drive actuators that are supported by four folded beam suspensions. Thus, the movable cavity can move in the lateral direction. Both cavity nanobeams are designed to be of 700 nm in width. There are 79 holes etched in each beam with a constant period of 310 nm, forming a PhC cavity. The diameters of the holes are quadratically tuned from 190 nm at the center to 40 nm at both ends^36^,^37^. The measured transmission spectrum of a typical PhC nanobeam photonic molecule when two cavities are perfectly aligned without lateral offset is shown in Fig. 1b, where resonance peaks of the transverse electric (TE)-like modes are marked (see Methods for the experiment setup). The interaction of the two cavities causes a splitting of their original uncoupled modes into corresponding pairs of even

-like and odd-like supermodes (If the two nanobeam cavities are not exactly identical due to microfabrication imperfections, the supermodes are not perfectly symmetric or anti-symmetric). The splitting is analogous to that of the electron states in diatomic molecules, where the degenerate atomic levels split into bonding and anti-bonding orbitals as a result of the strong coupling of two atoms^12^. In photonic molecules, the coupling strength of the modes is determined by the mode overlapping of the two uncoupled cavities, which is sensitive to the relative displacement between them^12^. In our work, when the movable cavity is displaced laterally, the coupling strength can be varied drastically from a negative to the positive sense and vice versa, resulting in the variation of the mode splitting width (see Fig. 1c).

### Analytical and numerical analysis of lateral tuning of PhC nanobeam photonic molecules

The mode coupling in a PhC nanobeam photonic molecule can be described by the coupled-mode theory^38^,^39^ (see Supplementary Note 1 for the detailed theoretical model). We first consider a PhC nanobeam photonic molecule, in which *ω*_1_ (*ω*_2_) and *τ*_1_ (*τ*_2_) are the mode frequencies and photon lifetimes of the high Q factor uncoupled PhC nanobeam cavities respectively. The eigenvalues 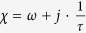 of the coupled supermodes are given by


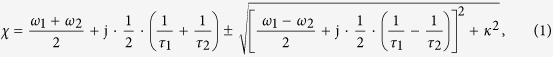


where *к* is the coupling strength between the two PhC nanobeam cavities, and is determined by^38^


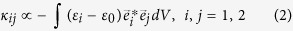


and the assumption *κ = κ*_*12*_ =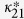 is utilized when the two modes couple in a loss-free way^38^. *ε*_*i*_ and *ε*_0_ are the permittivities for the cavity and its surrounding medium, respectively. 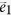 and 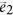 are the electric field profiles of the two modes. Obviously, the frequency difference between the two coupled supermodes (i.e. mode splitting width) Δ*ω* is mainly determined by the initial frequency detuning Δ = |*ω*_1_ − *ω*_2_| and coupling strength *к*. Besides, the mode parity can be obtained from the coupled-mode equations (see Supplementary Note 1 for the detailed theoretical model) and the phase difference between the two coupled modes is


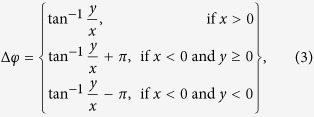


where *x*  = (*ω* − *ω*_2_)/*к*, and *y*  = (1/*τ*−1/*τ*_2_)/*к*. In this work, due to the high Q factors of the PhC nanobeam cavities, *y* is at least one order of magnitude smaller than *x*, and thus the value of 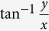 is close to zero. For *x *> 0, the electric fields in two coupled cavities are therefore in phase (Δ*φ* ≈ 0) and the corresponding coupled supermode is even-like. Otherwise for *x *< 0, (Δ*φ* ≈ ±π) and the coupled supermode is odd-like.

**Figure 2:**
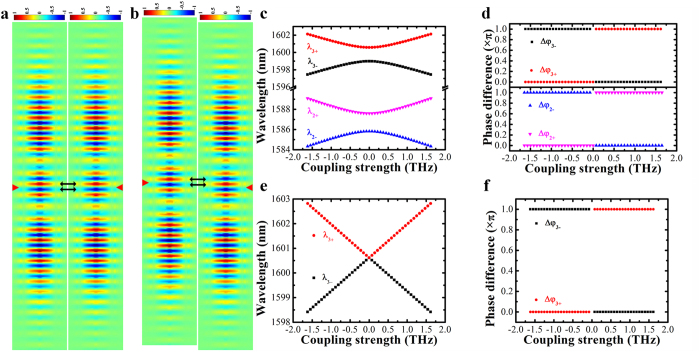
(**a)** At initial condition when the cavity holes are perfectly aligned, the overlapping electric fields of two single PhC nanobeam cavity modes are in-phase (the red arrow indicates the cavity center). Notes: The mode profile shows the in-plane electric field component (in the direction perpendicular to the nanobeam) in a horizontal plane at half the silicon nanobeam height, and the resonance wavelength is around 1600.5 nm; (**b**) When the movable PhC nanobeam cavity is laterally shifted by one lattice, the overlapping electric fields of two single cavity modes are out-of-phase (the red arrow indicates the cavity center); Supermode wavelengths (**c**) and phase differences Δ*φ* (**d**) vary as functions of coupling strength *κ* for non-zero detuning case; Supermode wavelengths (**e**) and phase differences Δ*φ* (**f**) vary as functions of coupling strength *κ* for zero detuning case. The results are calculated with the coupled-mode theory. Notes: the Q factors used for the calculation of (**c–f)** are set at 1×10^4^ which is a reasonable value according to our experiments, and the corresponding resonance wavelengths of uncoupled PhC nanobeam cavities can be found in (**c**,**e**) when coupling strength *κ* is zero.

In the NEMS tunable PhC nanobeam photonic molecule, as shown in Fig. 2a, the mode distributions of the two uncoupled cavities are originally symmetric about the perpendicular bisector plane of the line segment that connects the centers of the coupled cavities. Hence, the electric fields of both uncoupled cavities in the overlapping integration area have the same sign. According to Eq. (2), the coupling strength is negative. When the movable cavity is laterally shifted by one lattice, the signs of the electric fields of the two uncoupled cavities in the overlapping integration area are opposite to each other (shown in Fig. 2b). Therefore, the coupling strength becomes positive. It is this unique integrated NEMS tuning mechanism that allows the coupling strength to vary precisely from negative strong coupling to zero (weak coupling) and further to positive strong coupling with a lateral displacement about one PhC lattice.

We calculate the eigenwavelengths *λ*_±_ (with *λ*_+_ denoting a longer wavelength) of a pair of coupled supermodes as the coupling strength changes from a negative value to a positive value. For non-zero detuning (Δ = |*ω*_1_ − *ω*_2_| ≠  0), when *к* is negative and increases towards zero, *λ*_+_ decreases and *λ*_−_ increases, resulting in reduced mode splitting width as shown in Fig. 2c for the selected second and third order modes. Their respective phase differences Δ*φ* are calculated and shown in Fig. 2d. It is observed that the supermodes corresponding to *λ*_+_ and *λ*_−_ have even-like and odd-like parities, respectively. When *к* becomes zero, the mode splitting width is equal to the initial detuning (see Supplementary Note 1 for details). As *к* continues to increase from zero to a positive value, *λ*_+_ increases while *λ*_−_ decreases, and the mode splitting width increases. In addition, the supermode corresponding to *λ*_+_ now becomes an odd-like mode, whereas that corresponding to *λ*_−_ becomes an even-like mode, which can be concluded from the mode phase differences shown in Fig. 2d. The mode parity inversion is due to oscillation of the evanescent field along the lateral tuning direction that leads to the sign inversion of the coupling strength^20^,^21^,^31^,^32^,^33^,^34^,^35^. In the case of zero detuning (*ω*_1_ = *ω*_2_), the variation trends of *λ*_+_ and *λ*_−_ are similar to that of non-zero detuning, but the mode splitting vanishes when *к* is zero, as shown in Fig. 2e for the selected third order supermodes. As *к* increases from a negative value to a positive value, the supermode corresponding to *λ*_+_ (*λ*_−_) also changes from even-like (odd-like) mode to odd-like (even-like) mode, as indicated in Fig. 2f.

We also performed 3D-Finite Difference Time Domain (FDTD) simulations to calculate the supermode wavelength shifts and mode field distributions of the PhC nanobeam photonic molecule when the movable cavity is set at different lateral displacements. The FDTD method generates a time-domain pulse as the excitation source to the feeding waveguide. According to the Fourier transform theory, a time-domain confined signal spreads over a broad frequency range in the frequency-domain. We record the transmitted electromagnetic field in the time-domain. Subsequently, a Fourier transform reveals the frequency-domain response of the input pulse, which corresponds to the device transmission spectrum. The peaks within the forbidden band denote the supermodes, whose mode profiles, orders, and symmetries are then determined at their respective frequencies.

**Figure 3:**
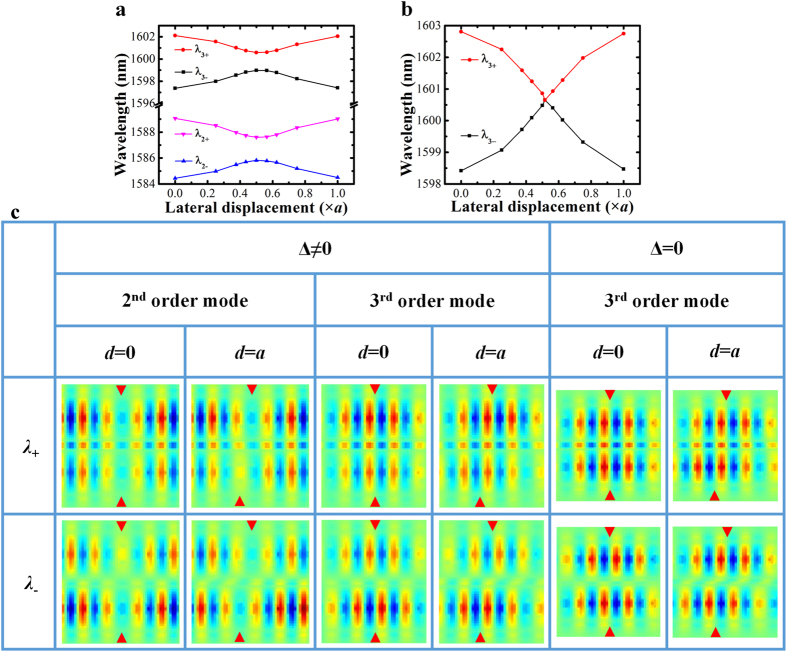
The 3D FDTD simulation results of PhC nanobeam photonic molecule for variation in lateral displacement. (**a**) Wavelength variations of the 2^nd^ order (*λ*_2±_) and the 3^rd^ order (*λ*_3±_) supermodes with respect to the increase in lateral displacement for non-zero detuning case; (**b**) Wavelength variations of the 3^rd^ order supermodes with respect to the increase in lateral displacement for zero detuning case; (**c**) Mode profile variations for both non-zero detuning (Δ  ≠  0) and zero detuning cases (Δ  = 0) with red arrows indicating cavity centers. Note: The lattice period of the two coupled PhC nanobeam cavities is fixed at 310 nm (*a*  = 310 nm) for both non-zero and zero detuning cases. The mode profile indicates the in-plane electric field component (in the direction perpendicular to the nanobeam) in a horizontal plane at half the silicon nanobeam height.

**Figure 4:**
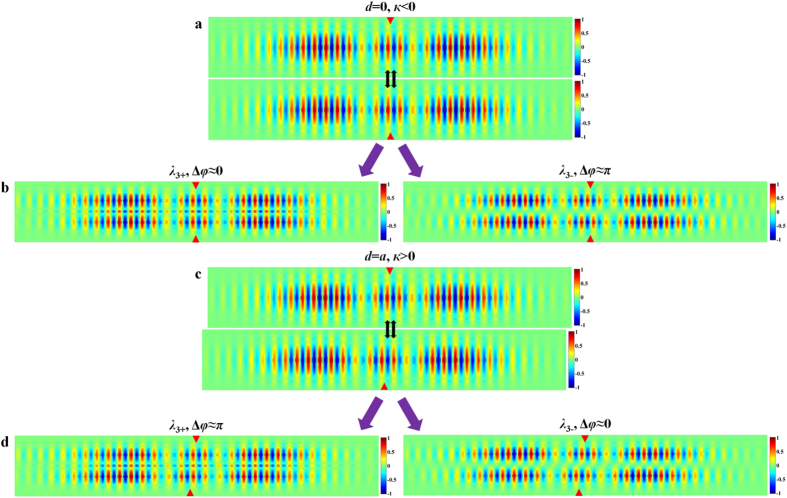
(**a)** The mode profiles of two uncoupled PhC nanobeam cavities with zero center-to-center lateral offset; (**b)** the FDTD simulated supermode profiles of PhC nanobeam molecule with no center-to-center lateral offset between two nanobeams; (**c**) the mode profiles of two uncoupled PhC nanobeam cavities with a lateral offset of one PhC lattice period *a* (*a*  = 310 nm); (**d**) the FDTD simulated supermode profiles of coupled PhC nanobeam cavities with a lateral center-to-center offset of 310 nm; Notes: the mode profile indicates the in-plane electric field component (in the direction perpendicular to the nanobeam) in a horizontal plane at half the silicon nanobeam height, and the resonance wavelength of each single cavity is around 1600.5 nm.

The zero detuning case is simulated with two identical cavities, while the non-zero detuning case is simulated with two cavities having slightly different nanobeam widths. The simulation results are summarized in Fig. 3. When the movable cavity is laterally shifted (see the schematics in Fig. 2a,b), the coupling strength increases from an initial negative value to zero at about half PhC lattice offset and then further increases to a positive value for one lattice offset. The FDTD simulated wavelength shifts of the selected supermodes, shown in Fig. 3a,b respectively for both non-zero detuning and zero detuning cases, are in good agreement with the results predicted with the coupled-mode theory shown in Fig. 2c,e (the relationships between the resonance mode wavelength and coupling strength corresponding to Fig. 3a,b are shown in Fig. S1a,b, respectively). Figure 3c further highlights the mode field distributions obtained from FDTD simulations for various cavity-coupling configurations with different lateral offsets *d*. To save space, only a central part of the field distribution is shown for each supermode with two red triangles indicating two cavity centers. These mode profiles shown in Fig. 3c also support the mode parity predictions obtained from the coupled-mode theory. To illustrate this clearly, we take the zero detuning case (*ω*_1_ = *ω*_2_ = *ω*_0_) for an example and consider two uncoupled third order modes from two identical single cavities that are put close to each other with zero center-to-center lateral offset (*d* = 0 nm), as shown schematically in Fig. 4a. The coupling strength is determined to be negative (*к* < 0) with Eq. (2). Subsequently, *x* = (*ω* − *ω*_0_)/*к* is positive for the supermode with a longer wavelength (*λ*_+_), and thus according to Eq. (3), the phase difference Δ*φ* is zero and the mode is even-like. On the contrary, the supermode having a shorter wavelength (*λ*_−_) has an odd-like parity with a phase difference Δ*φ* equal to π. Taking the original uncoupled mode profiles and their phase differences into consideration, the resulting supermode field distributions of the coupled cavities can be predicted conceptually by superposition and are confirmed by the FDTD simulated third order supermodes shown in Fig. 4b. Similarly, we consider the configuration where two cavities have a lateral center-to-center offset of one PhC lattice period (*d* = 310 nm). The uncoupled third order mode profiles and their relative positions are illustrated in the Fig. 4c. Clearly, the coupling strength is now positive (*к* > 0), and the mode parities are inversed. The supermode with a longer wavelength (*λ*_+_), having *x* = (*ω* − *ω*_0_)/*к* < 0 and thus Δ*φ* = π, becomes odd-like, whereas the supermode having a shorter wavelength (*λ*_−_) becomes even-like. Again taken the original uncoupled mode profiles shown in Fig. 4c and their phase differences in supermodes into consideration, the resulting field distributions of the coupled cavities can be deduced and match well with the FDTD simulated third order supermodes shown in Fig. 4d. The parity of a supermode can also be determined by comparing the signs of the electric fields at identical locations in two coupled cavities (see Fig. 3c). Taking the second order supermodes for example, at *d* = 0 nm, the sign of the electric field at each cavity center is the same for *λ*_+_ mode and different for *λ*_−_ mode, indicating their even-like and odd-like parity respectively. At *d* = 310 nm however, the sign of the electric field at each cavity center becomes different for *λ*_+_ mode and the same for *λ*_−_ mode instead, which clearly indicates a parity inversion.

### Lateral tuning of PhC nanobeam photonic molecules in experiments

**Figure 5:**
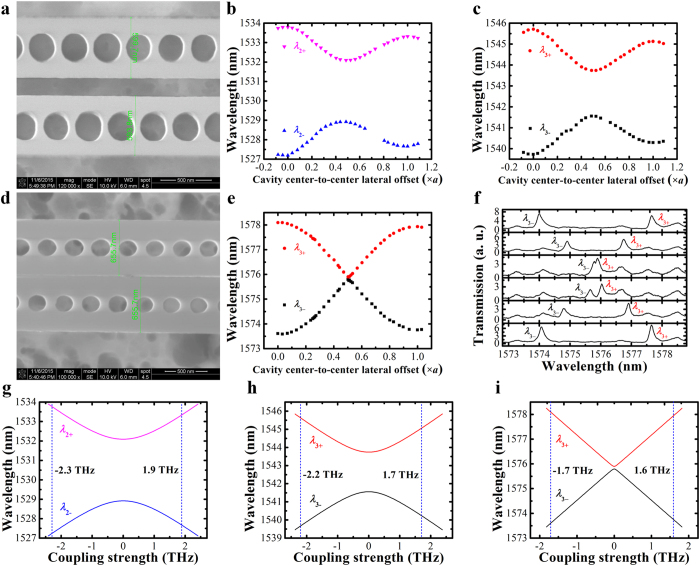
(**a)** SEM image showing the central part of a non-zero detuned PhC nanobeam photonic molecule (Sample A) with measured widths of the nanobeams annotated; (**b**,**c)** show respectively the measured wavelengths of the 2^nd^ order and 3^rd^ order supermodes as functions of the cavity center-to-center lateral offset for Sample A; (**d)** SEM image showing the central part of a nearly zero detuned PhC nanobeam photonic molecule (Sample B) with measured widths of the nanobeams annotated (Note: The movable nanobeam cavity accidentally collapsed to the fixed one after the experiment, causing an irreversible stiction of the two cavities); (**e**) Measured wavelengths of the 3^rd^ order supermodes as functions of the cavity center-to-center lateral offset for Sample B, with (**f)** showing its corresponding transmission spectra recorded at different cavity center-to-center lateral offsets (*d*  = 62 nm, 113 nm, 156 nm, 166 nm, 209 nm, and 257 nm, respectively from top to bottom); (**g,h**) show respectively the calculated wavelength variations of the 2^nd^ order and 3^rd^ order supermodes with respect to the increase in coupling strength for Sample A; (**i**) shows the calculated wavelength variations of the 3^rd^ order supermodes with respect to the increase in coupling strength for Sample B. Note: *a* is the lattice period of the PhC nanobeam cavities and is 310 nm.

In our experiments, the lateral displacement of the movable nanobeam cavity is precisely controlled by the integrated NEMS comb drive actuators. Through gradually raising the applied DC voltage, the lateral displacement is increased stepwise with 1~16 nm per step (see Supplementary Note 2 for the detailed lateral displacement calibration). The transmission spectra of PhC nanobeam photonic molecule under different cavity center-to-center lateral offsets are obtained (see Methods for the experiment setup). For non-zero detuning as shown in Fig. 5a (Sample A), the widths of the two PhC nanobeam cavities are measured to be around 594 nm and 600 nm under SEM, respectively. The resonance wavelengths of the second and third order supermodes at different cavity center-to-center lateral offsets are illustrated in Fig. 5b,c, respectively. Clearly, the mode splitting width decreases with increment in lateral offset until the mode splitting width reaches a minimum value, which is related to the initial frequency detuning of the two uncoupled cavities. As the lateral offset continues to increase, the mode splitting width increases again. Compared with the third order supermodes, the second order supermodes have a larger mode splitting minimum, which indicates a larger initial frequency detuning.

In our unique NEMS lateral tuning design here, both the mode frequencies (*ω*_1_, *ω*_2_) and photon lifetimes (*τ*_1_, *τ*_2_) of the uncoupled PhC nanobeam cavities can be experimentally obtained by Lorentzian fitting the measured resonance peaks at near-zero coupling strength at a lateral offset around a half of a PhC lattice where the mode splitting width reaches the minimum (see Supplementary Note 3 for details of the measurement). With *ω*_1_, *ω*_2,_ *τ*_1_, and *τ*_2_ known, the wavelengths of the PhC nanobeam photonic molecule supermodes at various coupling strength are calculated using Eq. (1) and plotted in Fig. 5g,h. Comparing the mode splitting widths in Fig. 5g to those in Fig. 5b, it can be concluded that the coupling strength *κ* is continuously tuned from −2.3 THz to 1.9 THz for the second order supermodes. Similarly, the experimental coupling strength tuning range is from −2.2 THz to 1.7 THz for the third order supermodes by comparing Fig. 5c–h.

Figure 5d further presents a nearly zero detuning case (Sample B), where both of the nanobeams are measured to be around 658 nm in width. Similarly, when the cavity center-to-center lateral offset increases, the mode splitting width decreases to a minimum and then increases again as predicted (see Fig. 5e,f). In this case, the minimum mode splitting width, which is equal to the initial mode detuning between the two uncoupled PhC nanobeam cavities, is only 80 GHz (Δ*λ*  ≈ 106 pm). Again, with the mode frequencies (*ω*_1_, *ω*_2_) and photon lifetimes (*τ*_1_, *τ*_2_) of the uncoupled PhC nanobeam cavities obtained (see Supplementary Note 3 for details of the measurement), the wavelengths of the photonic molecule supermodes as functions of the coupling strength are computed and plotted in Fig. 5i. Comparing it to the experimental results in Fig. 5e, we observe that the coupling strength *κ* is continuously tuned from −1.7 THz to 1.6 THz. Finally, it should be noted that, by comparison, it is not possible to change the sign of the coupling strength with the traditional coupling gap tuning approach 24,25,40 (see Supplementary Note 4 for details of the coupling gap tuning approach).

**Figure 6:**
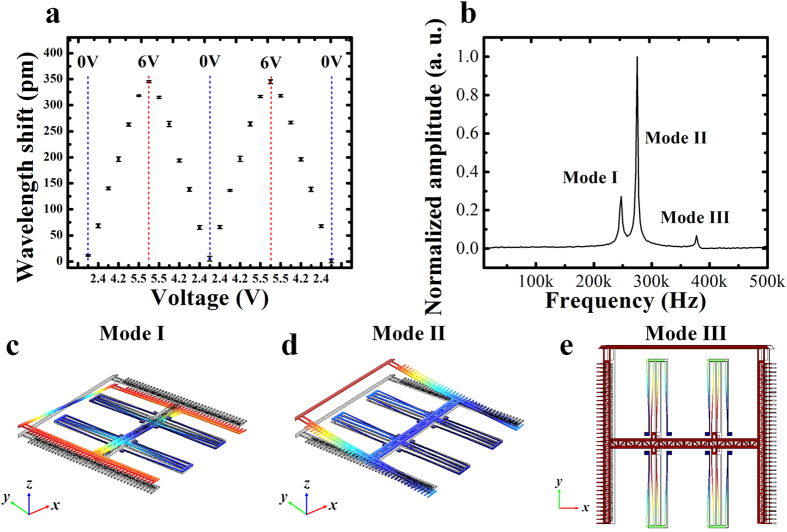
(**a**) Mode wavelength shifts with repeated variations of applied voltage on the NEMS actuator. The error bar at every voltage value indicates the standard deviations of mode wavelength, obtained by repeating measurements four times. (**b**) Measured frequency response of the NEMS actuator. The lowest three mechanical vibration modes are observed, which are respectively denoted by Mode I (248 kHz), Mode II (275 kHz), and Mode III (378 kHz). The simulated mode shapes using FEM are shown in (**c**) for Mode I, (**d**) for Mode II, and (**e**) for Mode III, respectively.

Besides having a relatively large range from negative to zero and further to positive, the tuning of the coupling strength in our device is also reversible. To demonstrate this, the applied voltage on the NEMS actuator is repeatedly tuned upwards and downwards, and the corresponding resonance wavelength shifts are obtained. As shown in Fig. 6 for example, the wavelength of the second order *λ*_−_ supermode exhibits first a red shift when the applied voltage increases, and returns to the initial value when the voltage reverts to zero. The maximum wavelength standard deviation recorded at each voltage is less than 4.8 pm (i.e. the standard deviation of mode splitting width Δ*ω* is less than 3.8 GHz), which indicates that the coupling strength standard deviation is less than 1.9 GHz (|*к*| ≈ Δ*ω*/2 and see Supplementary Note 1 for the relationship between mode splitting width and coupling strength). Therefore, the integrated NEMS actuator is able to control the coupling strength of the PhC nanobeam photonic molecule with a precision of less than 2 GHz over a range of several THz in our experiments.

Dynamic performance of the proposed coupling strength control of the PhC nanobeam photonic molecule is determined by its integrated NEMS actuator, whose frequency response is measured and shown in Fig. 6b (see Methods for details of the experimental setup). Three lowest mechanical vibration modes of the actuator are experimentally recorded in the frequency range from 200 kHz to 400 kHz. They are identified and match well with the simulation results using finite element method (FEM). According to the simulated mode shapes shown in Fig. 6c–e, Mode I is the fundamental vibration mode of the NEMS actuator at 248 kHz and Mode II is the second order mode at 275 kHz. They are out-of-plane twisting modes about the *y* and *x* axes, respectively. Mode III is the third order vibration mode at 378 kHz and is an in-plane translational mode that leads to the lateral displacement of the movable cavity along the *x* axis. These results indicate that the coupling strength of our PhC naobeam photonic molecules can be dynamically controlled with a fast response time at microsecond level. Further reducing the response time is possible through NEMS structural designs for example by decreasing actuator size and increasing suspension spring constant.

## Discussion

In conclusion, we have experimentally demonstrated the precise control of the coupling strength in PhC nanobeam photonic molecules using integrated NEMS. The coupling strength can be dynamically adjusted with a precision of a few GHz over a range of several THz without altering the nature of their constituent cavities. Moreover, both negative and positive coupling strengths are experimentally realized. The control of coupling strength in photonic molecules is crucial both in the fundamental study of cavity quantum electrodynamics and the development of efficient quantum information devices^32^. Our work paves a way to optimize the coupling strength in photonic molecules *in situ*, which provides a feasible platform for the study of the strong interaction in quantum emitters embedded photonic molecule system^18^. By precisely tuning the coupling strength of a photonic molecule, one is able to control the interaction between the quantum emitters that are individually embedded in the two nanocavities of the photonic molecule and thus the entanglement generation^13^,^41^,^42^. In single photon devices that are based on photonic molecules, the single photon statistics can be improved by tailoring the coupling strength^14^. The Spontaneous symmetry breaking (SSB) in photonic molecules, which is quite sensitive to the coupling strength, can also be achieved with our proposed method^15^. Moreover, the fine tuning of coupling strength may also facilitate the study of Josephson physics in a photonic molecule^16^,^17^.

## Methods

### Fabrication of NEMS controlled PhC nanobeam molecules

The device is fabricated on a silicon-on-insulator (SOI) wafer having a 260 nm-thick top silicon (Si) device layer. Electron beam lithography (EBL) is used to form the patterns of the PhC nanobeam cavities, NEMS actuators, and nanowire waveguides, which are sequentially fully etched up to the buried oxide (BOX) layer using deep reactive-ion-etching (DRIE). Next, another EBL step is used to form the patterns of grating couplers and tapered rib waveguides. These patterns are shallowly etched to 80 nm with DRIE. The isolation trenches and electrode bonding pads are fabricated through a series of standard photolithography, DRIE, Au deposition with electron beam evaporator, and lift-off processes. The whole wafer is subsequently diced into small chips. The final process is the HF vapor release to free the suspended structures from the substrate. Before testing, the chip is packaged with a dual in-line package (DIP) and the pins are electrically connected to the device using wire bonding.

### Experimental setup

The device is tested with a tunable laser source (TLS) with wavelengths ranging from 1520 nm to 1620 nm and an optical spectrum analyzer (OSA). The light from the TSL is conducted through a single-mode (SM) fiber and coupled into the tapered rib waveguide by the input grating coupler, which is designed to allow only TE-like mode light to be coupled into the waveguide. The grating coupler has a period of 600 nm and filling ratio of 280:320. The total area of the coupler is about 12 × 12 μm^2^. The rib waveguide is tapered from 12 μm at the grating coupler to 700 nm at the cavity beam. Next, the light is coupled out from the PhC nanobeam photonic molecule to another rib waveguide to the output grating coupler and is finally collected by a multi-mode (MM) fiber. The output light is detected and monitored by the OSA. To measure the frequency response of the integrated NEMS actuator, a network analyzer is employed to scan the frequency of an AC voltage with fixed amplitude applied to the NEMS actuator, and the laser wavelength is fixed at the half maximum position of a selected PhC photonic molecule resonance mode. The transmitted light intensity is detected by a photodetector and its electric output signal is monitored by the network analyzer.

## Additional Information

**How to cite this article**: Du, H. *et al.* Precise control of coupling strength in photonic molecules over a wide range using nanoelectromechanical systems. *Sci. Rep.* **6**, 24766; doi: 10.1038/srep24766 (2016).

## Supplementary Material

Supplementary Information
