# Supplementary material for: Precise control of coupling strength in photonic molecules over a wide range using nanoelectromechanical systems
Source: Sci Rep. 2016 Apr 21;6:24766. doi: 10.1038/srep24766 (PMC4838863; doi:10.1038/srep24766)
Supplement: Supplementary Information [file srep24766-s1.pdf]

Precise control of coupling strength in photonic molecules over a wide range using nanoelectromechanical systems

Han Du[1,+], Xingwang Zhang[1,+], Guoqiang Chen[1], Jie Deng[2], Fook Siong Chau[1], Guangya Zhou[1,*]

[1] *Department of Mechanical Engineering, National University of Singapore, 9 Engineering Drive 1, Singapore 117576*

[2] *Institute of Materials Research and Engineering, A*STAR (Agency for Science, Technology, and Research), 2 Fusionopolis Way, Innovis, #08-03, Singapore 138634*

[+] *These authors contributed equally to this work*

[*] *mpezgy@nus.edu.sg*

Supplementary Note 1: Coupled-mode theory for photonic molecules

The mode coupling in a photonic crystal (PhC) nanobeam photonic molecule can be described by coupled-mode theory[1, 2]. We first consider a PhC nanobeam photonic molecule, in which ω_1 (ω_2) and τ_1 (τ_2) are the resonance frequencies and photon lifetimes of the uncoupled high Q factor PhC nanobeam cavites, respectively. The mode amplitude in one cavity is denoted by A_1, and the mode amplitude in the other cavity is denoted by A_2. Then we have the following coupled-mode equations:

$$\frac{dA_1}{dt} = j\omega_1 A_1 - \frac{1}{\tau_1} A_1 + j\kappa_{12} A_2 \tag{S-1}$$

$$\frac{dA_2}{dt} = j\omega_2 A_2 - \frac{1}{\tau_2} A_2 + j\kappa_{21} A_1 \tag{S-2}$$

where, κ_{12} and κ_{21} are the coupling strengths between the two PhC nanobeam cavities. Consider a coupled supermode at frequency χ, i.e. $A_1=a_1\, exp(j\chi t)$ and $A_2=a_2\, exp(j\chi t)$, based on Eqs. (S-1) and (S-2), we have the matrix equation

$$\begin{bmatrix} j\omega_1 - \frac{1}{\tau_1} & j\kappa_{12} \\ j\kappa_{21} & j\omega_2 - \frac{1}{\tau_2} \end{bmatrix} \cdot \begin{bmatrix} a_1 \\ a_2 \end{bmatrix} = j\chi \cdot \begin{bmatrix} a_1 \\ a_2 \end{bmatrix}, \tag{S-3}$$

To have non-trivial solutions of a_1 and a_2, the following determinant must be zero.

$$\begin{vmatrix} j\omega_1 - \frac{1}{\tau_1} - j\chi & j\kappa_{12} \\ j\kappa_{21} & j\omega_2 - \frac{1}{\tau_2} - j\chi \end{vmatrix} = 0. \tag{S-4}$$

So, the eigenvalues ($\chi = \omega + j \cdot \frac{1}{\tau}$) of the coupled supermodes are given by

$$\chi = \frac{\omega_1+\omega_2}{2} + \mathrm{j} \cdot \frac{1}{2} \cdot \left(\frac{1}{\tau_1} + \frac{1}{\tau_2}\right) \pm \sqrt{\left[\frac{\omega_1-\omega_2}{2} + \mathrm{j} \cdot \frac{1}{2} \cdot \left(\frac{1}{\tau_1} - \frac{1}{\tau_2}\right)\right]^2 + \kappa^2}, \tag{S-5}$$

where κ_{12} and κ_{21} are determined by[1]

$$\kappa_{ij} \propto -\int (\varepsilon_i - \varepsilon_0) \vec{e}_i^{\,*} \vec{e}_j dV,\ i, j=1, 2 \tag{S-6}$$

and we assume $\kappa = \kappa_{12} = \kappa_{21}^*$ when the two modes couple in a loss-free way[1]. ε_i and ε_0 are the permittivities for cavity and the surrounding medium, respectively. $\vec{e}_1$ and $\vec{e}_2$ are the electric field profiles of the two modes. When the frequency detuning is zero (i.e. $\omega_1=\omega_2=\omega_0$), Eq. (S-5) becomes

$$\chi = \omega_0 + \mathrm{j}\cdot\frac{1}{2}\cdot\left(\frac{1}{\tau_1}+\frac{1}{\tau_2}\right) \pm \sqrt{\kappa^2 - \left[\frac{1}{2}\cdot\left(\frac{1}{\tau_1}-\frac{1}{\tau_2}\right)\right]^2}. \tag{S-7}$$

Clearly, if $|\kappa| > \frac{1}{2}\cdot\left|\frac{1}{\tau_1}-\frac{1}{\tau_2}\right|$, the mode split into two modes, which corresponds to the strong coupling regime. If $|\kappa| \leq \frac{1}{2}\cdot\left|\frac{1}{\tau_1}-\frac{1}{\tau_2}\right|$, the mode splitting vanishes, which corresponds to the weak coupling regime. When the two modes have the same Q factor ($\tau_1 = \tau_2$), according to Eq. (S-7), the absolute value of coupling strength is equal to a half of mode splitting width (i. e. $|\kappa| = \Delta\omega/2$).

The parities of the coupled modes can be obtained from Eqs. (S-3) and (S-5)

$$\frac{a_1}{a_2} = \frac{j\cdot(\omega-\omega_2)-(\frac{1}{\tau}-\frac{1}{\tau_2})}{j\cdot\kappa} = \frac{\omega-\omega_2}{\kappa} + j\cdot\frac{\frac{1}{\tau}-\frac{1}{\tau_2}}{\kappa}. \tag{S-8}$$

According to Eq. (S-8), the phase difference $\Delta\varphi$ between a_1 and a_2 is

$$\Delta\varphi = \begin{Bmatrix} \tan^{-1}\frac{y}{x}, & \text{if } x > 0 \\ \tan^{-1}\frac{y}{x}+\pi, & \text{if } x < 0 \text{ and } y \geq 0 \\ \tan^{-1}\frac{y}{x}-\pi, & \text{if } x < 0 \text{ and } y < 0 \end{Bmatrix}, \tag{S-9}$$

where

$$x = \frac{\omega-\omega_2}{\kappa},\ y = \frac{\frac{1}{\tau}-\frac{1}{\tau_2}}{\kappa}. \tag{S-10}$$

In the experiment, due to the high Q factor of the PhC nanobeam cavity, $\frac{1}{\tau}-\frac{1}{\tau_2}$ is at least one order smaller than ω-ω_2. Therefore, $\tan^{-1}\frac{y}{x}$ is close to zero. According to Eq. (S-9), when $x > 0$, $\Delta\varphi \approx 0$, which indicates the sign of the electric field in both of the cavity is the same, and the

corresponding mode is even-like mode. Otherwise, when $x < 0$, $\Delta\varphi \approx \pm\pi$, the sign of the electric field in both of the cavity is opposite, and the corresponding mode is odd-like mode.

When the coupling strength (κ) is zero, Eq. (S-5) becomes,

$$\chi_1 = \omega_1 + \mathrm{j} \cdot \frac{1}{\tau_1}, \tag{S-11}$$

$$\chi_2 = \omega_2 + \mathrm{j} \cdot \frac{1}{\tau_2}. \tag{S-12}$$

In order words, the resonance frequencies and photon lifetimes of the PhC nanobeam photonic molecule supermodes are equal to those of the uncoupled PhC nanobeam cavities, i.e. (ω_1, ω_2) and (τ_1, τ_2) respectively, when the coupling strength is zero. The mode splitting width is thus equal to the initial frequency detuning ($\Delta = |\omega_1 - \omega_2|$).

Supplementary Note 2: Lateral displacement calibration

The electrostatic comb drive and folded beam suspensions are standard designs. The electrostatic force generated by the comb drive is given by

$$\mathrm{F} = n\varepsilon t V^2/g, \tag{S-13}$$

where n is the number of movable fingers, ε is the permittivity, t and g are the finger thickness and finger gap spacing, and V is the applied voltage. The spring constant of a single set of the folded beam suspension is given by

$$\mathrm{k} = b^3 t E/l^3, \tag{S-14}$$

where b, and l are the flexural beam width and length respectively, and E is the Young's Modulus of the beam material. Consequently, the displacement d can be obtained as

$$\mathrm{d} = \Phi V^2, \tag{S-15}$$

$$\Phi = n\varepsilon l^3/(mb^3 gE), \tag{S-16}$$

where m is number of folded beam suspensions and Φ denotes a constant. Therefore, the lateral displacement of the movable beam is linearly proportional to the square of applied voltage.

At initial zero center-to-center offset between two cavities, the mode splitting width of a pair of photonic molecule supermodes is at maximum. When the movable beam is laterally shifted, the mode splitting width of the photonic molecule is changed. When the lateral displacement is equal to one lattice period (a = 310 nm) of the PhC nanobeam cavity, the absolute value of coupling strength reaches a maximum again, so does the mode splitting width. In our experiment, we gradually increase the applied voltage and record those (V_1 and V_2) at two consecutive mode splitting maxima, where the corresponding lateral displacement difference is equal to 310 nm. According to Eq. (S-15), the value of Φ can be obtained with Φ = 310 nm / (V_2^2-V_1^2), and the

lateral displacements, or equivalently the cavities' center-to-center offsets, under various applied voltages are calculated and listed in the Table S1 below.

The coupling strength at every FDTD simulated center-to-center offset between two cavities is further calculated based on Eq. (S-5), and the relationship between the FDTD simulated resonant mode wavelength and coupling strength is plotted below.

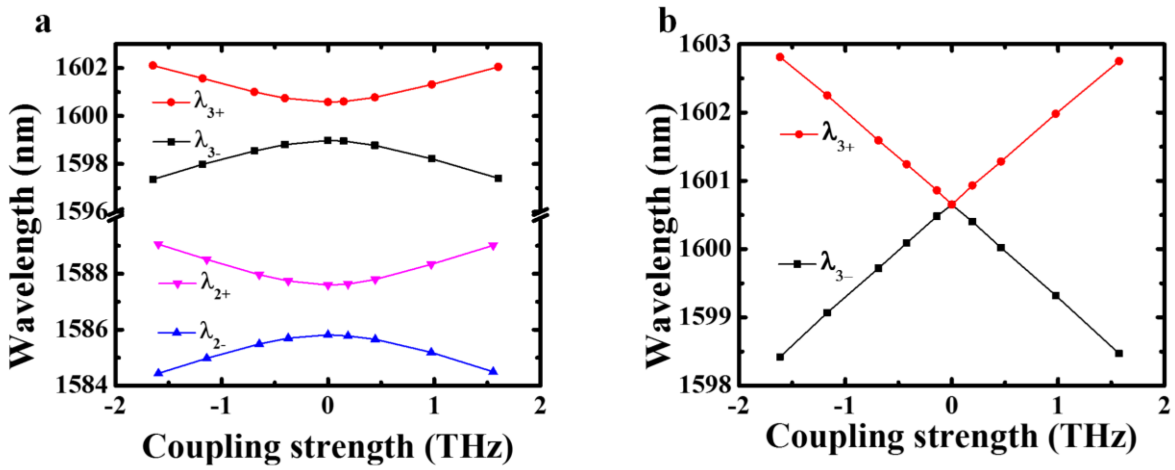

Figure S1: the relationship between the resonance mode wavelength and coupling strength. (a) The non-zero detuning ($\Delta \neq 0$) case corresponding to Fig. 3a; (b) the zero detuning case ($\Delta = 0$) corresponding to Fig. 3b.

Table S1: the cavity center-to-center lateral offset s of various applied voltages

Sample A (Δ≠0)			Sample B (Δ≈0)		
**Voltage squared (V^2)**	**Cavity center-to-center lateral offset (nm)**	**Cavity center-to-center lateral offset (×*a*)**	**Voltage squared (V^2)**	**Cavity center-to-center lateral offset (nm)**	**Cavity center-to-center lateral offset (×*a*)**
**0**	-25.48	-0.08219	**0**	0	0
**5.76**	-13.50	-0.04355	**5.76**	9.94	0.03208
**12.25***	0*	0*	**12.25**	21.15	0.06822
**17.64**	11.21	0.03616	**17.64**	30.45	0.09824
**24.01**	24.46	0.07890	**24.01**	41.45	0.13372
**30.25**	37.44	0.12077	**30.25**	52.22	0.16847
**36**	49.40	0.15935	**36.00**	62.15	0.20049
**42.25**	62.40	0.20129	**42.25**	72.94	0.23530
**47.61**	73.55	0.23725	**44.89**	77.50	0.25000
**53.29**	85.36	0.27536	**46.24**	79.83	0.25752
**59.29**	97.84	0.31562	**47.61**	82.20	0.26515
**65.61**	110.99	0.35802	**49.00**	84.60	0.27289
**72.25**	124.80	0.40258	**53.29**	92.00	0.29678
**77.44**	135.59	0.43740	**59.29**	102.36	0.33020
**84.64**	150.57	0.48571	**65.61**	113.27	0.36539
**90.25**	162.24	0.52335	**72.25**	124.74	0.40237
**96.04**	174.28	0.56220	**77.44**	133.70	0.43128
**102.01**	186.70	0.60225	**79.21**	136.75	0.44113
**108.16**	199.49	0.64352	**84.64**	146.13	0.47137
**114.49**	212.66	0.68599	**90.25**	155.81	0.50262
**121**	226.20	0.72967	**91.2025**	157.46	0.50792
**125.44**	235.43	0.75946	**92.16**	159.11	0.51325
**132.25**	249.60	0.80515	**94.09**	162.44	0.52400
**136.89**	259.25	0.83629	**96.04**	165.81	0.53486
**144**	274.04	0.88399	**102.01**	176.11	0.56811
**148.84**	284.10	0.91647	**108.16**	186.73	0.60236
**156.25**	299.52	0.96618	**114.49**	197.66	0.63761
**161.29**	310	1	**121.00**	208.90	0.67387
**169**	326.04	1.05173	**125.44**	216.56	0.69860
**174.24**	336.94	1.08689	**132.25**	228.32	0.73652
			**136.89**	236.33	0.76236
			**144.00**	248.61	0.80196
			**148.84**	256.96	0.82892
			**156.25**	269.76	0.87018
			**161.29**	278.46	0.89825
			**169.00**	291.77	0.94119
			**174.24**	300.82	0.97037
			**179.56**	310	1
			**184.96**	319.32	1.03007

*Due to fabrication imperfections, the movable cavity of Sample A has an initial lateral offset (see Fig. 5b and 5c). The cavity center-to-center lateral offset is zero when the voltage squared is 12.25 V^2

Supplementary Note 3: Estimation of the resonance frequencies and photon lifetimes of the uncoupled PhC nanobeam cavities

According to the analysis in Supplementary Note 1, the resonance frequencies and photon lifetimes of a pair of PhC nanobeam photonic molecule supermodes are equal to the corresponding modes of the uncoupled PhC nanobeam cavities when the coupling strength is zero. In our experiment, we increase the applied voltage until the mode splitting width reaches a minimum, where the coupling strength is zero and the corresponding device transmission spectra are recorded and shown in Fig. S2, from which the resonance wavelengths and Q factors are obtained through Lorentzian fitting the resonance peaks. Subsequently, the resonance frequencies (ω_1, ω_2) and photon lifetimes (τ_1, τ_2) of the uncoupled PhC nanobeam cavities are calculated and the results are listed in Table S2 ($\omega = 2\pi c/\lambda$, $\tau = 2Q/\omega$).

As shown in Fig. 1a, there are two waveguides feeding into cavity-1 and cavity-2, respectively. These two waveguides are close and thus not completely decoupled (similarly for the two output waveguides). When the PhC photonic molecule is excited from the upper waveguide that is on cavity-1 side, a portion of light can also couple into the waveguide that is on cavity-2 side. The excited cavity-2 resonance can also couple back into the output waveguide in a similar way. In other words, both cavity-1 and cavity-2 are excited and detected in our experiment even when the coupling strength of the two cavities is 0.

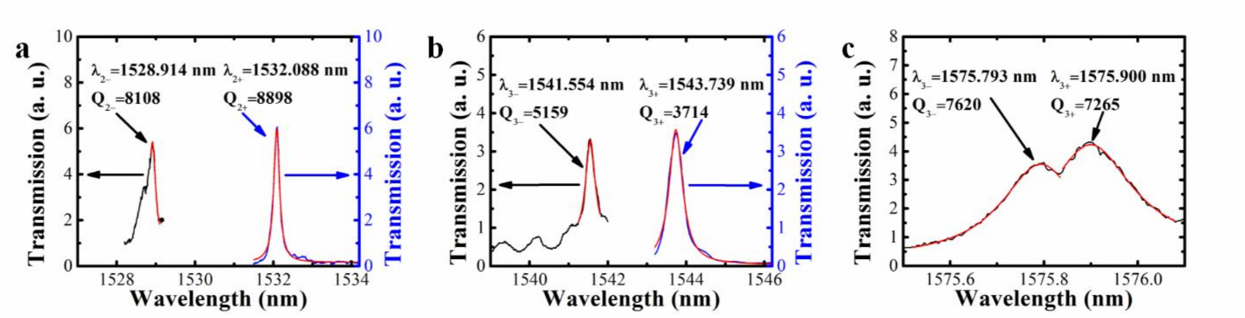

Figure S2: Transmission spectra of Sample A (a, b) and Sample B (c) when the coupling strength is tuned to zero. The modes are fitted with Lorentz profile (red curves). (a) The second order modes of Sample A; (b) the third order modes of Sample A; (c) the third order modes of Sample B.

Table S2: the resonance frequencies (ω_1, ω_2) and photon life time (τ_1, τ_2) in the uncoupled PhC nanobeam cavites

Sample NO.	Mode order	ω_1 (THz)	ω_2 (THz)	τ_1 (ps)	τ_2 (ps)
A(Δ≠0)	2nd	1232.872	1230.318	13.153	14.465
	3rd	1222.763	1221.033	8.438	6.083
B(Δ≈0)	3rd	1196.195	1196.114	12.740	12.148

Supplementary Note 4: Coupling gap tuning of photonic molecules

For traditional coupling gap tuning of PhC nanobeam photonic molecules, the cavities' center-to-center lateral offset is kept at zero, while the gap between the two cavities varies. We calculate the resonance wavelength shifts and mode field distribution variations of a pair of supermodes of the PhC nanobeam photonic molecule when the coupling gap increases using 3D Finite Difference Time Domain (FDTD) simulations. As shown in Fig. S3a, the strong coupling between the two PhC nanobeam cavities leads to a mode splitting, and the splitting width decreases as the gap increases. When the gap is large enough (> 500 nm), the mode splitting disappears, corresponding to the weak coupling regime. During the coupling gap tuning process, the even mode always has a longer wavelength while the odd mode always has a shorter wavelength, which indicates that the sign of the coupling strength and mode parity do not inverse. (see Fig. S3b). Furthermore, in order to tune the coupling strength to zero, the coupling gap has to increase by more than 400 nm, which requires a much larger displacement compared with our lateral tuning method (~150 nm).

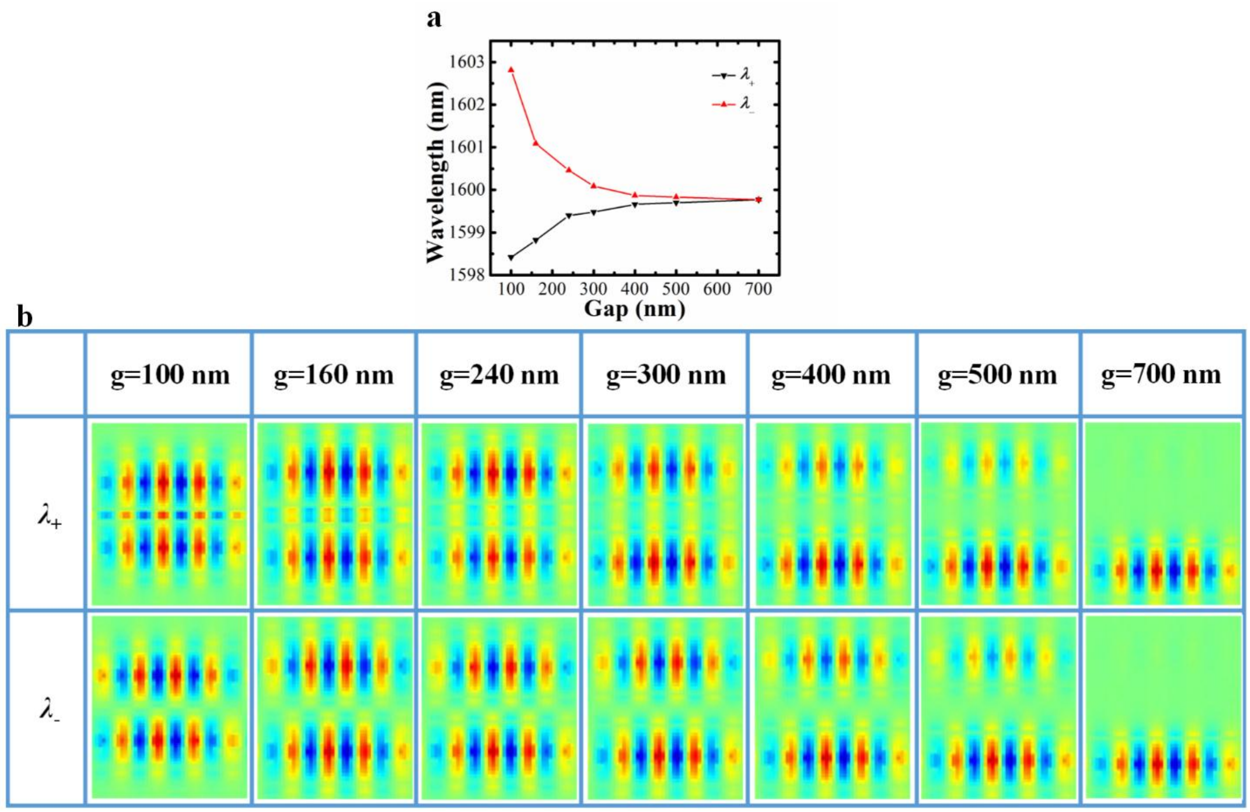

Figure S3: 3D FDTD simulation results of coupling gap tuning of PhC nanobeam photonic molecules: (a) resonance wavelength variations of a pair of supermodes as functions of the gap; and (b) corresponding supermode profiles at different coupling gaps. Notes: the mode profile indicates the in-plane electric field component (in the direction perpendicular to the nanobeam) in a horizontal plane at half the silicon nanobeam height.

References

1. Hermann A. Haus WH. Coupled-mode theory. *Proc IEEE* **79**, 1505-1518 (1991).

2. Lin H, Chen JH, Chao SS, Lo MC, Lin SD, Chang WH. Strong coupling of different cavity modes in photonic molecules formed by two adjacent microdisk microcavities. *Opt Express* **18**, 23948-23956 (2010).
